# Pretreatment on *Miscanthus lutarioriparious* by liquid hot water for efficient ethanol production

**DOI:** 10.1186/1754-6834-6-76

**Published:** 2013-05-10

**Authors:** Hong-Qiang Li, Cheng-Lan Li, Tao Sang, Jian Xu

**Affiliations:** 1 National Key Laboratory of Biochemical Engineering, Institute of Process Engineering, Chinese Academy of Sciences, Beijing, 100190, People’s Republic of China; 2 Key Laboratory Plant Resources and State Key Laboratory of Systematic & Evolutionary Botany, Institute of Botany, Chinese Academy of Sciences, Beijing, 100093, People’s Republic of China

**Keywords:** *Miscanthus lutarioriparious*, Liquid hot water pretreatment, Simultaneous saccharification and fermentation, Ethanol

## Abstract

**Background:**

The C4 perennial grass *Miscanthus giganteus* has proved to be a promising bio-energy crop. However, the biomass recalcitrance is a major challenge in biofuel production. Effective pretreatment is necessary for achieving a high efficiency in converting the crop to fermentable sugars, and subsequently biofuels and other valued products.

**Results:**

*Miscanthus lutarioriparious* was pretreated with a liquid hot water (LHW) reactor. Between the pretreatment severity (PS) of 2.56-4.71, the solid recovery was reduced; cellulose recovery remained nearly unchanged; and the Klason lignin content was slightly increased which was mainly due to the dissolving of hemicellulose and the production of a small amount of pseudo-lignin. The result shows that a LHW PS of 4.71 could completely degrade the hemicellulose in *Miscanthus*. Hemicellulose removal dislodged the enzymatic barrier of cellulose, and the ethanol conversion of 98.27% was obtained.

**Conclusions:**

Our study demonstrated that LHW served as an effective pretreatment in case that *Miscanthus lutarioriparious* was used for ethanol production by simultaneous saccharification and fermentation. The combination and the pretreatment method of *Miscanthus* feedstock holds a great potential for biofuel production.

## Background

As a C4 perennial plant characterized with high biomass yield and relatively low nitrogen and water requirement, *Miscanthus* is considered to be one of the top candidates of second-generation energy crops [1,2]. The previous study showed that carbohydrates in *Miscanthus giganteus* constituted approximately 75% of its dry matter content [2]. Recently, some investigations have been published on the pretreatment and enzymatic hydrolysis of *Miscanthus*. The treatments employed included ammonia fiber expansion (AFEX) [3], NaOH pretreatment [4], organic acid [5], organosolv [6], wet explosion [7] and liquid hot water (LHW) pretreatment [8].

To improve the rate of enzyme hydrolysis and increase yields of fermentable sugars from carbohydrates, all kinds of pretreatment methods, such as dilute acid [9,10], steam explosion [11-14], lime [15], wet oxidation [7,16], AFEX [17-19], alkali [20-25], SPORL [26,27], organosol [6], microwave [28,29], ozone and LHW [30-32] have been developed. LHW pretreatment uses water as the thermal medium. Compared to other technologies, LHW pretreatment has the following advantages: no extra chemicals added, no special non-corrosive materials requirement for reactor building, and fewer toxic degradation products formed [33]. It was considered as an environmentally friendly method, and has now become an attractive process for lignocellulosic biomass pretreatment [34].

In addition, most of the investigations on *Miscanthus* merely evaluated the pretreatment effects on its enzymatic hydrolysis, with only a few on ethanol production [4]. The ultimate objective of the pretreatment is to convert biomass into the final products such as ethanol or butanol. Because some fermentation inhibitors toxic to the microorganisms might be formed in the pretreatment [35], a good enzymatic digestibility does not mean that the fermentable sugars generated by the enzymatic hydrolysis can be smoothly converted to ethanol. Simultaneous saccharification and fermentation (SSF) is a more appropriate way to evaluate the pretreatment performance [36].

This study evaluated the feasibility and efficiency of LHW pretreatment on *Miscanthus lutarioriparious*, a promising energy crop in China [37,38], for ethanol production. The harvested *Miscanthus lutarioriparious* contained a certain amount of soil, sand and other impurities. In order to remove these impurities, the *Miscanthus lutarioriparious* was washed by tap water. After washing, the suspended and precipitated part was collected separately. To investigate the influence of the pretreatment on different fraction and the subsequent bioconversion process, the two parts were subjected to LHW pretreatment and following SSF. The solid recovery after LHW pretreatment, the potential inhibitors formed, and ethanol conversion from LHW-pretreated *Miscanthus* were investigated. In this work, the probable LHW pretreatment severity range required of *Miscanthus* was obtained and verified the adaptability of LHW on Miscanthus. The ethanol yield of *Miscanthus* was obtained and compared to other raw materials pretreated with different methods.

## Results and discussion

### *Miscanthus* component analysis

**Figure 1:**
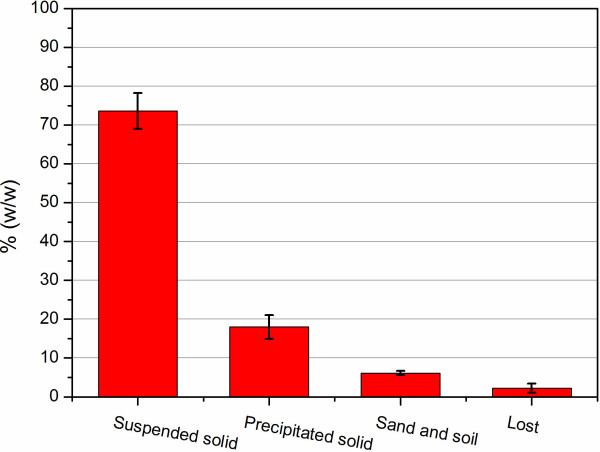
**Mass percentage of different parts separated from *Miscanthus *by water-washing.**

Tap water was used to wash off the impurity in the raw *Miscanthus lutarioriparious* to avoid interference in the subsequent component analysis and bioconversion. After water-washing, both the suspended and precipitated parts were collected separately. Figure 1 shows the mass (dry weight) percentage of different parts of *Miscanthus*. The suspended solid made up about 3/4 of the whole raw material, and the content of sand and soil was more than 5%. In the washing process, about 2.22% loss on the biomass and water-soluble sugars was observed, which was relatively low compared to the water-extractable ingredients in the corn stover [39].

**Figure 2:**
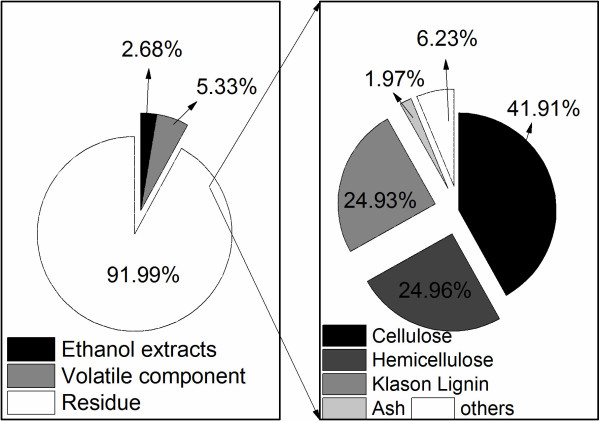
**Composition of suspended solid of *Miscanthus*.**

Figure 2 shows the composition of the suspended solid of *Miscanthus*. Cellulose was the predominant component accounting for 41.2% of the residue after the ethanol extraction. The content of hemicellulose and Klason lignin was very close. The paired *t*-test of Minitab 16 (Minitab Inc.) software was used to compare the composition difference of suspended and precipitated solids (data not shown). The *t*-value was 0, and the P-value was 1, which indicated that there was no significant difference in the composition between the two solids at the significance level of 0.01.

### The effect of LHW on solid recovery and *Miscanthus* chemical composition

Both the suspended and the precipitated parts were pretreated with LHW. The solid recovery (SR) at different pretreatment severity (PS) is listed in Table 1. There was a similar variation trend found for SR for both the suspended and precipitated parts. As PS increased, SR gradually reduced. For the suspended part, 95.00% of the solid were recovered at PS of 2.65. It dropped to 65.46% when PS increased to 4.71. Similarly, SR of 76.05%-64.85% was obtained for the precipitated part as PS changed from 3.53 to 4.71. The reduction of SR was mainly caused by the hydrolysis of hemicellulose in the pretreatment process which can be seen from Table 1. There was no hemicellulose found for the suspended and precipitated parts when PS was 4.71 or 4.12. This implied that submerging the *Miscanthus* into water completely maybe get a better LHW pretreatment effect. Furthermore, the optimal PS may be between 4.12 and 4.71. A further PS optimization may be needed. The enzymatic hydrolysis efficiency of cellulose in lignocellulosic material is influenced by many factors. The hemicellulose’s shielding effect to cellulase has been recognized as one of the key factors affecting the cellulose digestion [40-42]. The acidic environment formed under the LHW pretreatment condition contributed to the hemicellulose hydrolysis. The acetic acid produced from hemicellulose acetyl hydrolysis further promoted the hemicellulose hydrolysis [43]. In general, a lower hemicellulose content means a higher cellulose enzymatic hydrolysis efficiency. Therefore, the removal ratio of the hemicellulose is employed as a key indicator to evaluate a pretreatment technology. Compared with the hemicellulose, the cellulose and lignin are not easily hydrolyzed in the conventional LHW pretreatment process, and they tend to remain in the solid. This caused the increase on the mass percentage of the cellulose and lignin in the pretreated biomass. In addition, some pseudo-lignin might be produced. The pseudo-lignin can be broadly defined as an aromatic material that yields a positive Klason lignin value. However, it is formed by the combination of carbohydrate and lignin degradation products, not from the native lignin. It has been reported that pseudo-lignin was produced in some pretreatment process, especially in the dilute acid pretreatment [44,45]. In the present study with LHW pretreatment, the suspended solid had Klason lignin recovery ratios of 104.87%, 104.95 and 107.69% under PS of 2.65, 3.53 and 4.71, respectively (Table 1).

**Table 1:** **The composition and composition recovery of *Miscanthus *after LHW pretreatment**

**Samples**	**PS**	**Solid recovery %**	**Composition, %**				**Composition recovery, %**		
			**Cellulose**	**Hemicellulose**	**Klason lignin**	**Ash**	**Cellulose**	**Hemicellulose**	**Klason Lignin**
Suspended solid	0	-	39.21 ± 1.80	23.47 ± 1.74	21.36 ± 1.42	2.87 ± 0.07	-	-	-
	2.65	95.00 ± 1.60	40.47 ± 1.29	20.34 ± 1.76	23.58 ± 1.99	1.76 ± 0.13	98.05	82.33	104.87
	3.53	88.19 ± 1.25	43.41 ± 0.00	19.94 ± 0.00	25.42 ± 0.00	1.88 ± 0.05	97.64	86.46	104.95
	4.12	70.74 ± 1.88	45.89 ± 2.25	10.03 ± 0.57	29.67 ± 0.46	1.85 ± 0.20	82.79	35.58	98.26
	4.71	65.46 ± 0.16	55.00 ± 1.11	0.00 ± 0.00	35.14 ± 0.01	2.21 ± 0.10	91.82	0	107.69
Precipitated solid	0	-	42.20 ± 2.08	22.99 ± 2.26	21.23 ± 0.17	4.67 ± 0.72	-	-	-
	3.53	76.05 ± 1.22	46.03 ± 0.49	10.15 ± 0.25	28.52 ± 0.35	1.45 ± 0.11	82.95	33.58	102.16
	4.12	68.24 ± 0.23	53.54 ± 1.68	0.00 ± 0.00	30.36 ± 0.32	1.82 ± 0.01	86.58	0	97.59
	4.71	64.85 ± 0.46	60.97 ± 0.00	0.00 ± 0.00	31.24 ± 1.01	2.09 ± 0.36	93.69	0	95.43

### Degradation products in the LHW pretreatment

**Figure 3:**
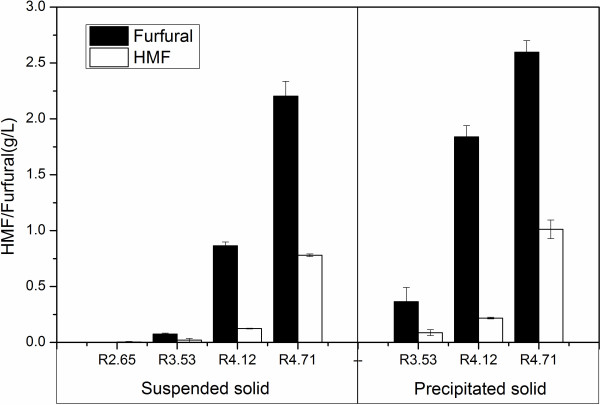
HMF and furfural formed in LHW pretreatment.

In the pretreatment with higher temperature, hexose and xylose can be further degraded to 5-hydroxyl furfural (HMF) and furfural [46]. Figure 3 shows the HMF and furfural formed in the present investigation from different PS. Obviously, both HMF and furfural yield increased as PS increased. However, furfural yield was always much higher than HMF with the same pretreatment condition. For the suspended part, the furfural and HMF concentrations in the hydrolysate were 0.075 and 0.021 g/L with PS at 3.53. When PS was increased to 4.71, they were 2.2 and 0.78 g/L, respectively. Under the harshest severity of 4.71, furfural and HMF concentrations from the precipitated part were 2.6 and 1.0 g/L. If the hydrolysates are used to product ethanol, furfural and HMF in the hydrolysate will inhibit the growth of yeast and decrease ethanol yield and productivity. As volatile inhibitory compounds, furfural and HMF can be removed by heating and vaporization to alleviate the toxic effect [46].

**Figure 4:**
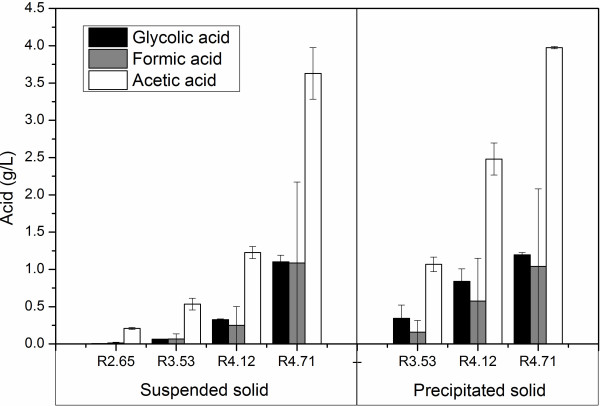
Organic acids formed in LHW pretreatment.

In addition to HMF and furfural, glycolic, formic and acetic acid were also found in the pretreatment hydrolysate which is presented in Figure 4. Clearly, the formation of all these acids followed the similar trend, i.e., the concentrations increased as PS increased. During the pretreatment process, the deacetylation of acetyl groups present in the hemicellulose led to the formation of the acetic acid [47]. The acetic acid concentration reached 3.6 and 4.0 g/L for the suspended and precipitated hydrolysates, respectively, with PS at 4.71. Formic acid can be formed when HMF is broken down [46]. The formation of glycolic acid was due to the breakdown of glucose and xylose [48]. Compared to the acetic acid, the concentrations of formic and glycolic acid were much less, not more than 1.5 g/L. The toxic effect of aliphatic acid on *Saccharomyces cerevisiae* is attributed to the undissociated form. Formic acid (pKa = 3.75) has a higher toxic effect than glycolic acid (pKa = 3.90) and acetic acid (4.76). However, inhibition of yeast was found to be apparent at their concentrations exceeding 100 mM and lower concentrations than 100 mM gave higher ethanol yields than fermentations with no aliphatic acids included [46]. In this work, the aliphatic acids produced during LHW pretreatment were lower than 100 mM, and consequently beneficial for the ethanol yield rather than harmful.

An optimal pretreatment condition is needed to consider the use of cellulose, hemicellulose and lignin at the same time. However, if these components require different pretreatment conditions, there is no choice but to emphasize the main component. For example, cellulose was gained more attention in the present study. Under this pretreatment condition, a better cellulose enzymatic hydrolysis performance can be obtained, but part of hemicellulose and cellulose was degraded to furfural and HMF.

### SSF

**Figure 5:**
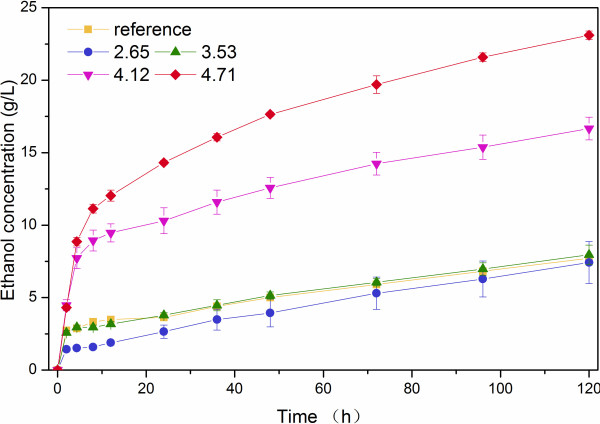
**SSF on suspended solid of *Miscanthus *with LHW pretreatment.**

**Figure 6:**
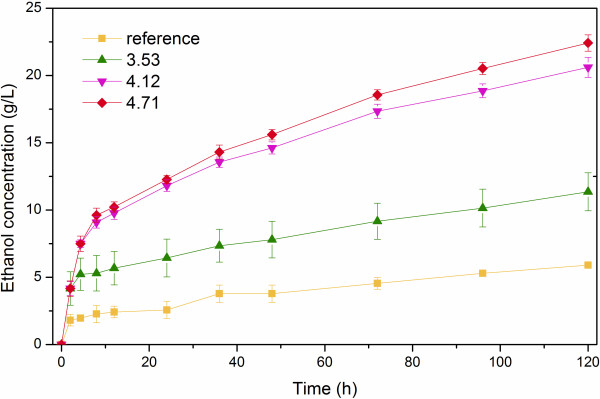
**SSF on precipitated solid of *Miscanthus *with LHW pretreatment.**

The SSF was carried out on the LWH pretreated *Miscanthus* and the untreated was used as reference. The ethanol concentrations were presented in Figures 5 and 6. The ethanol conversion was calculated based on Eq. (2). The ethanol conversion ratio for both the suspended and the precipitated part of *Miscanthus* gradually increased as PS increased. However, a lower PS was not distinct in improving the ethanol conversion. In the pretreatment on the suspended part with PS at 2.65, there was almost no appearance change found for the samples before and after pretreatment. The ethanol concentration from the pretreated suspended part and the untreated was 7.42 ± 1.45 g/L and 7.73 ± 0.21 g/L, respectively. The One-tailed Two Independent Samples Student's *t*-Test was used to compare these two results. The P value was 0.815. This indicates that there was no significant difference between the two results within the 95% confidence interval. Therefore, the PS of 2.65 was not utilized in the subsequent pretreatment on the precipitated part. For the suspended part with PS at 3.53, the ethanol conversion only increased by 13.7% compared to that from the raw suspended without pretreatment. When PS was increased to 4.12, the ethanol conversion improved dramatically and reached 84.87%. The highest ethanol conversion was found to be 98.27% as PS was at 4.71. This indicates that PS needs to be above some critical point for this *Miscanthus* feedstock to improve the biomass conversion effectively. In addition, such a higher conversion also showed that there were no toxic compounds produced in the SSF process. The similar trend of the ethanol conversion was observed on the precipitated part. With PS of 4.12, the ethanol conversion of the precipitated (89.86%) was more than three times compared to that from the raw precipitated part (26.34%).

At PS of 4.71, the ethanol conversion with the two parts separated by washing the raw *Miscanthus* showed almost no difference. However, at PS 4.12, the precipitated part had a better performance. Which implied that submerging the *Miscanthus* into water completely during LHW pretreatment maybe reduce the required energy consumption by using a lower PS in the *Miscanthus* biorefinery.

### Ethanol conversion

**Figure 7:**
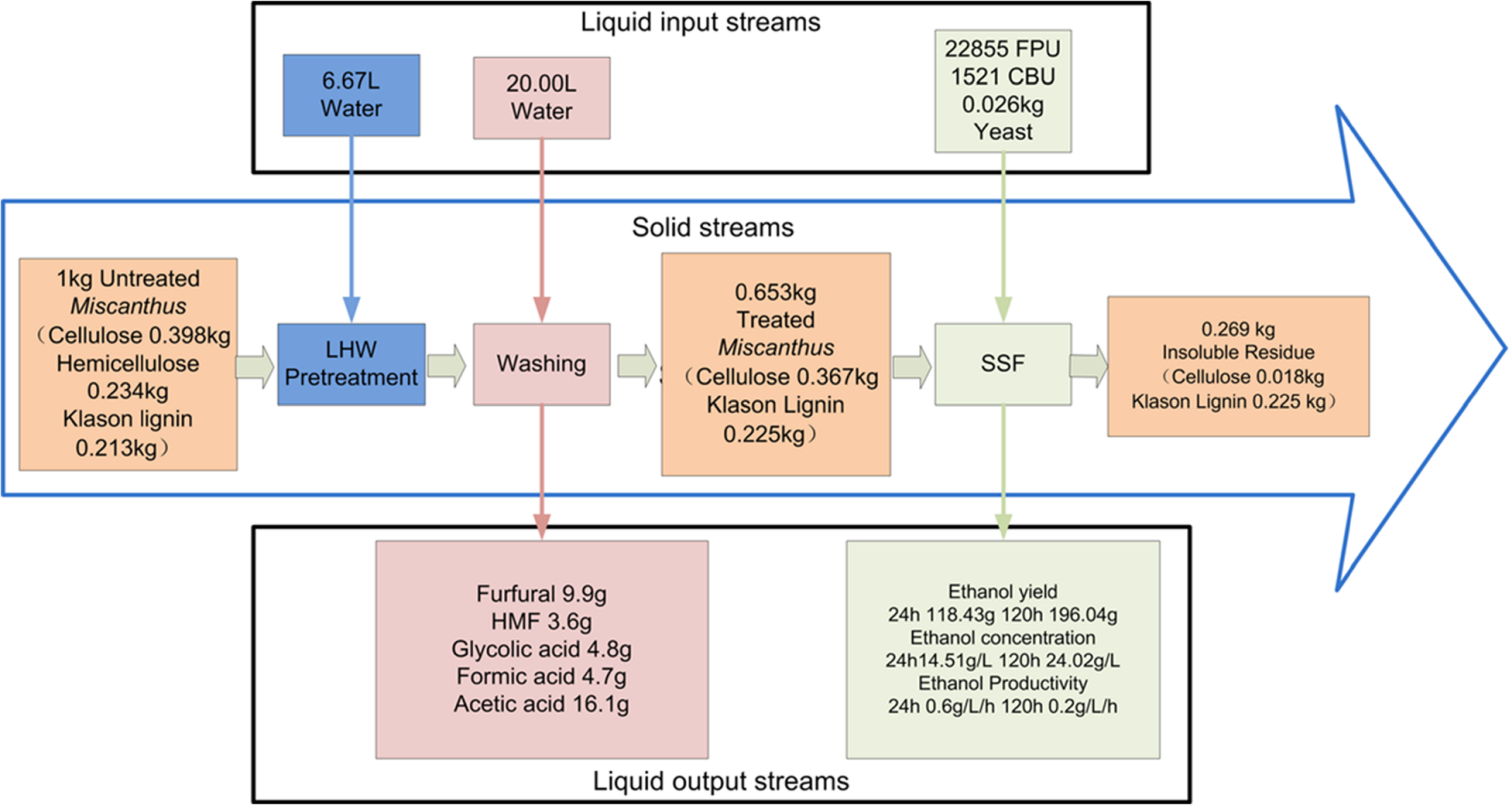
Mass balance analysis of Miscanthus LHW pretreatment and SSF process.

The ethanol conversions from the suspended and precipitated under PS of 4.71 are listed in Table 2. Based on Eq. (3), the ethanol conversion from *Miscanthus* pretreated with LHW was 14.72 g/100 g feedstock, more than twice higher than that from the untreated *Miscanthus* (7.12 g/100 g feedstock). Based on the PS of 4.71, Figure 7 gave a mass balance analysis of *Miscanthus* with LHW pretreatment and SSF. Table 3 lists the ethanol conversions from all kinds of biomass with different pretreatment methods. Compared with other investigations on *Miscanthus* pretreated by AFEX, ozone, etc., the present study gave a higher ethanol conversion. We also noted that the cellulase dosage loading used in this work was higher than some new reports [26]. It is maybe related to the fibers hornification due to sample drying. Hornification will lead to the collapse of the pores formed during pretreatment and affect the saccharification of pretreated samples [55,56]. Appropriate sample preparation is likely to reduce cellulase loading significantly. This indicates the LHW pretreatment is promising in pretreating *Miscanthus* to improve its conversion ratio efficiently.

**Table 2:** **Ethanol yield from *Miscanthus *with the pretreatment intensity of 4.71**

**Feedstock**	**Mass percentage based on the raw material (%)**	**Solid recovery after pretreatment (%)**	**Ethanol yield (g/100 g pretreated feedstock)**	**Ethanol yield (g/100 g raw feedstock)**
Raw Miscanthus	100	N.A	N.A.	7.12
The suspended	73.67	65.46	24.83	16.25
The precipitated	17.98	64.85	23.61	15.31

**Table 3:** Comparison on ethanol yield from different biomass

**Biomass**	**Pretreatment**	**Ethanol yield (g/g raw biomass)**	**References**
Corn straw	LHW, Lime, Wet oxidation, Steam explosion	0.02-0.10	[11,15,16,30]
Wheat straw	LHW, Steam explosion, Dilute acid	0.06-0.20	[9,12,31]
Rice straw	Dilute acid, Microwave with/without Alkali, Ammonia soaking	0.11	[10,20,21,28]
Sweet sorghum	Dilute ammonia, AFEX, Steam explosion	0.19	[13,17,22]
Switchgrass	Sodium hydroxide, AFEX, Hydrothermolysis	0.12-0.14	[18,19,23,32]
Sugarcane bagasse	Phosphoric acid pretreatment	0.25-0.27	[49]
Lodgepole pine	SPORL pretreatment	0.22	[26]
Aspen	Steam explosion, Dilute acid, Delignification	0.10	[14,24,27]
Birch	Alkaline	0.11	[25]
Spruce	Alkaline	0.08	[25]
Miscanthus	AFEX, Ethanol organosol, Microwave, Dilute acid pre-soaking, Ozone	0.10-0.11	[3,6,7,29,50]
Miscanthus	LHW	0.15	This study

## Conclusions

The feasibility of LHW pretreated *Miscanthus* used as ethanol production by SSF was studied. The result shows that a PS of 4.71 in LHW process could completely degrade the hemicellulose in *Miscanthus*. Hemicellulose removal improved the enzymatic digestibility of cellulose and obtained a higher ethanol bioconversion ratio of the theoretical. These results indicated that the LHW pretreatment was suitable for *Miscanthus* feedstock, which offer a promising solution to the biorefinery of this energy crop.

## Methods

### *Miscanthus*

The material of *Miscanthus lutarioriparious* used in this work was harvested in December, 2011 in Honghu County of the Hubei province, China. The air-dried raw material was cut into 1–2 cm and washed off the impurities including soil and sands. The *Miscanthus* was then divided into two parts according to the density difference: the suspended and the precipitated parts. To study the impact of the raw material density on *Miscanthus* bioconversion, these two portions were collected separately and used as the parallel raw materials for the subsequent experiments.

### Chemicals

Sulphuric acid (H_2_SO_4_, 98%) was purchased from Beijing Chemical Factory. Glucose, xylose, arabinose, Glycolic acid, formic acid, acetic acid, 5-hydroxymethyl furfural (HMF) and furfural were purchased from Sigma-aldrich, Co., (3050 Spruce Street, St Louis, Missouri 63103, US) as standards in HPLC analysis.

### Liquid hot water pretreatment

The LHW pretreatment was carried out in a laboratory made reactor. The hydrothermal reactor has a volume of 1000 mL equipped with a mechanical stirrer and cooling coils inside for a better temperature control. After the raw material mixed with water was put into the reactor, it was immersed into a salt bath with the temperature pre-set at 230°C. The PS was calculated with the following formula [51,52]:

(1) $$R_{O}=\int_{t_{1}}^{t_{2}}\exp\left(\frac{T-100}{14.75}\right)\;dt$$

**Figure 8:**
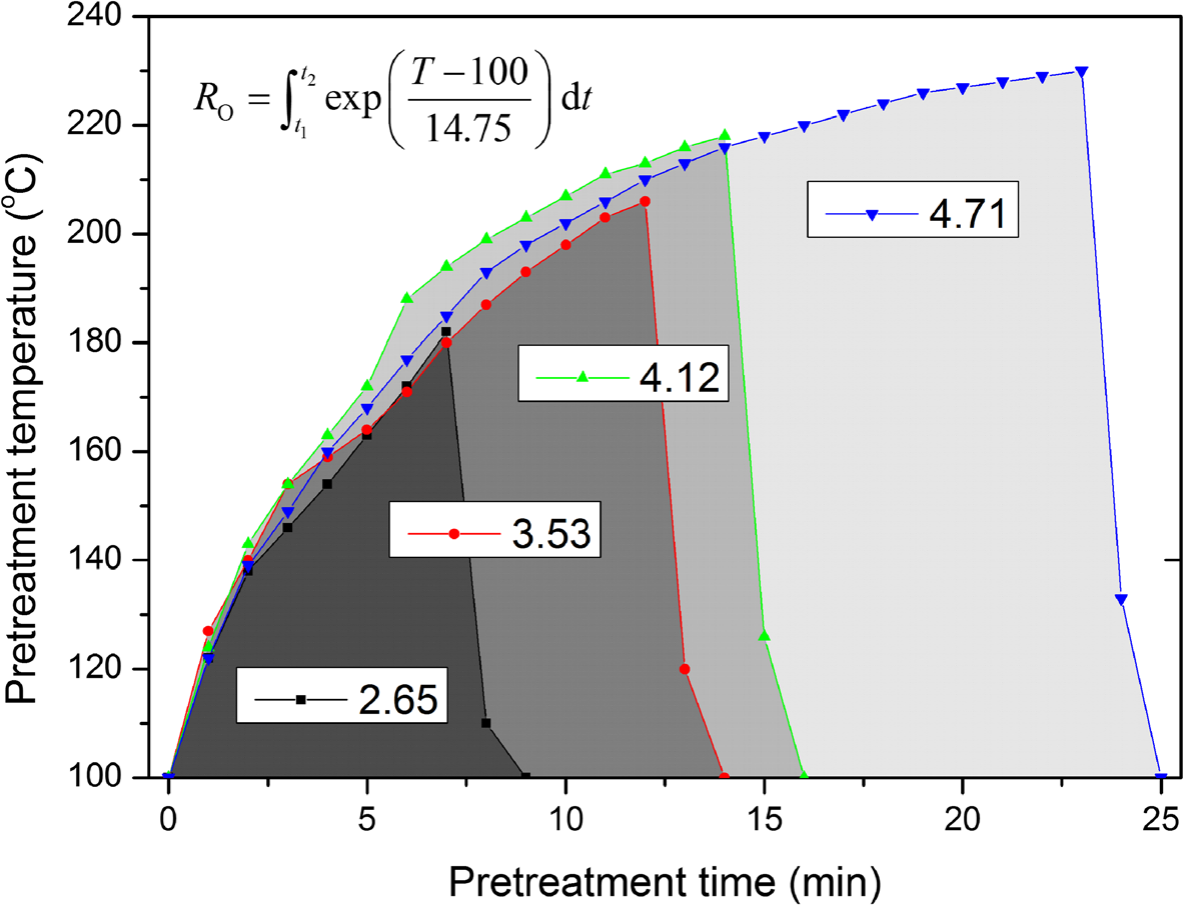
Correlation of pretreatment severity with pretreatment temperature and time.

*t*_1_ is the time at which the reactor temperature reaches 100°C and *t*_*2*_ is the time at which the reactor temperature drops to 100°C, (min), and *T* is the reactor temperature (°C). Figure 8 shows the correlation of the PS with the reaction temperature and time. When the pre-set PS was reached, cooling water was passed into the cooling coils. At the same time, the reactor was raised from the salt bath and put into a cooling water bath to rapidly cool the reactor, the water temperature in the reactor reduced to below 80°C within 2 minutes. When the temperature below 80°C, the reactor was opened to remove the pretreated material and end the pretreatment process. The liquid–solid ratio used in this study was 100:15 (V:W), the pre-set PS were 2.65, 3.53, 4.12 and 4.71. The slurry obtained from pretreatment was separated by vacuum filtration to get the hydrolyzed solution and water insoluble solid fraction. Solid was washed with 20-time water and dried at 60°C. The air-dried solid was used in the subsequent SSF. The hydrolysate volume and degradation products content were measured using HPLC.

### Compositional analysis of solid fraction

The composition of the pretreated as well as untreated Miscanthus was analyzed following NREL analytical procedures. According to the NREL LAP [53], a two-step sulfuric acid hydrolysis process was adopted to break down the structural polysaccharides into sugar monomers for quantification by HPLC. The acid-insoluble lignin (Klason lignin) residue was determined gravimetrically by subtracting the ash content from the residual obtained from the acid hydrolysis. All experiments were performed in duplicate, and the average value was calculated and presented.

### Monosaccharides and degradation products quantification

The monosaccharides from sulfuric acid hydrolysis and degradation products in LHW hydrolysate were determined by HPLC (Agilent 1260 Infinity, USA) with Hi-Plex H column (300 × 7.7 mm, Varian, Inc., Shropshire, UK) at 65°C with 5 mM H_2_SO_4_ as eluent at a flow rate of 0.6 mL min^-1^. A refractive index detector (RID) and a diode array detector (DAD) were used in series. The RID was used to determine the contents of glucose, xylose, arabinose, glycolic acid, formic acid and acetic acid. The DAD was used for HMF and furfural with a detection wavelength of 280 nm and a reference wavelength of 360 nm.

### SSF

The pretreatment effect of LHW was evaluated with SSF. The process was modified from the NREL’s procedure [54]. Three grams (dry weight) of treated and untreated material together with 32.30 mL citric acid buffer (pH = 4.8) and 2.2 mL cellulase (SUKAHAN(Weifang) Bio-Technology CO.,LTD) mixture solution (15FPU/g biomass and 1 CBU/g biomass) were placed into a 150-mL blue cap bottle. The pre-enzymatic hydrolysis was then carried out at 120 rpm, 50°C. After 24 hrs, the bottles were taken out and placed into an ice-water bath. The supplementation of 75 μL urea (24%, w/v), 2.93 mL cellulase solution (20 FPU/g biomass and 1.33 CBU/g biomass) and inoculated with 0.12 g Angel® thermal tolerance alcohol active dry yeast (TH-AADY, Product code: 80000012, Angel Yeast Co., Ltd., Yichang, China) was made to each bottle. The yeast is specifically selected high-quality alcohol yeast resistant to high temperature, alcohol and acid (http://angelyeast.en.alibaba.com/product/832168424-218419497/Angel_Thermal_Tolerance_Alcohol_Active_Dry_Yeast.html). The final solid biomass concentration was 8% (w/v). Nitrogen was used to replace the air in the bottles, and then the bottles were equipped with fermentation locks pre-filled with glycerol. The SSF was done at 42°C. At a certain time, the bottles were taken out and weighted for weight reduction calculating. The conversion of cellulose to ethanol was calculated as follows:

(2) $$\mathrm{Cellulose}\;\mathrm{conversion}=\frac{1.045\times C_{L}}{\frac{0.51\times C_{M}\times C_{C}}{0.9}}\times 100$$

1.045: Conversion factor of CO_2_ to ethanol;

0.51: Conversion factor of glucose to ethanol;

0.9: Conversion factor of cellulose to glucose.

The conversion of raw *Miscanthus* to ethanol was calculated as follows:

(3) $$\begin{array}{c} \mathrm{Miscanthus}\;\mathrm{conversion}=P_{S}\times R_{S}\times Y_{\mathrm{ES}}+P_{P}\\ \;\times R_{P}\times Y_{\mathrm{EP}} \end{array}$$

## Abbreviations

LHW: Liquid hot water; PS: Pretreatment severity; SSF: Simultaneous saccharification and fermentation; SR: Solid recovery; HMF: 5-hydroxyl furfural; TH-AADY: Thermal tolerance alcohol active dry yeast; CL: Weight reduction caused by CO_2_ emission, g; CM: Mass of sample used in SSF, g; CC: Mass percentage of cellulose in the sample, %; PS: Mass percentage of the suspended part after water-washing, %; RS: Cellulose recovery ratio of the suspended part after LHW pretreatment, %, it was 100 for the unpretreated sample; YES: Conversion of cellulose to ethanol on the suspended part, %; PP: Mass percentage of the suspended part after water-washing, %; RP: Cellulose recovery ratio of the precipitated part after LHW pretreatment, %, it was 100 for the unpretreated sample; YEP: Conversion of cellulose to ethanol on the precipitated part, %

## Competing interests

The authors declare that they have no competing interests.

## Authors' contributions

LH analyzed the data. LC-L carried out the pretreatment experiments and analysis. ST completed Miscanthus sample collection and characterization. XJ designed the project, supervised the experiments, interpreted the data and finalized the manuscript. All authors read and approved the final manuscript.
